# Past, Present and Future Distributions of an Iberian Endemic, *Lepus granatensis*: Ecological and Evolutionary Clues from Species Distribution Models

**DOI:** 10.1371/journal.pone.0051529

**Published:** 2012-12-13

**Authors:** Pelayo Acevedo, José Melo-Ferreira, Raimundo Real, Paulo Célio Alves

**Affiliations:** 1 Biogeography, Diversity and Conservation Research Team, University of Malaga, Malaga, Spain; 2 CIBIO, Centro de Investigação em Biodiversidade e Recursos Genéticos/InBio Laboratório Associado, Universidade do Porto, Vairão, Portugal; 3 Instituto de Investigación en Recursos Cinegéticos (CSIC-UCLM-JCCM), Ciudad Real, Spain; 4 Departamento de Biologia, Faculdade de Ciências, Universidade do Porto, Porto, Portugal; 5 University of Montana, Wildlife Biology Program, College of Forestry and Conservation, Missoula, Montana, United States of America; Consiglio Nazionale delle Ricerche (CNR), Italy

## Abstract

The application of species distribution models (SDMs) in ecology and conservation biology is increasing and assuming an important role, mainly because they can be used to hindcast past and predict current and future species distributions. However, the accuracy of SDMs depends on the quality of the data and on appropriate theoretical frameworks. In this study, comprehensive data on the current distribution of the Iberian hare (*Lepus granatensis*) were used to i) determine the species’ ecogeographical constraints, ii) hindcast a climatic model for the last glacial maximum (LGM), relating it to inferences derived from molecular studies, and iii) calibrate a model to assess the species future distribution trends (up to 2080). Our results showed that the climatic factor (in its pure effect and when it is combined with the land-cover factor) is the most important descriptor of the current distribution of the Iberian hare. In addition, the model’s output was a reliable index of the local probability of species occurrence, which is a valuable tool to guide species management decisions and conservation planning. Climatic potential obtained for the LGM was combined with molecular data and the results suggest that several glacial refugia may have existed for the species within the major Iberian refugium. Finally, a high probability of occurrence of the Iberian hare in the current species range and a northward expansion were predicted for future. Given its current environmental envelope and evolutionary history, we discuss the macroecology of the Iberian hare and its sensitivity to climate change.

## Introduction

Studying the distribution of a species and estimating its ecogeographical predictors are very important issues in ecology, macroecology and biogeography. The distribution range of a species is a complex expression of its ecology and evolutionary history, which is determined by diverse factors operating at different scales [1], [2]. In a conceptual framework, the presence of a species in a location at a given time, i.e., its distribution range, is mainly modulated by abiotic conditions (e.g., climatic or topographic variables), biotic factors (e.g., resources and presence of competitors) and by its ability to disperse [3]. Currently, techniques are available to elucidate the interaction between these factors within a mathematical framework, and therefore to empirically study the correlates of species distributions [4]. These techniques are known as species distribution models (SDMs), habitat suitability models, habitat distribution models or niche models [5].

SDMs are increasingly being used to address a diverse range of practical questions in ecology. For example, they are used to determine the macroecological requirements of singular species [6], to suggest unsurveyed sites with a high potential for the occurrence of rare [7] and/or invasive species [8], to infer the biogeographical relationships of ecologically related species [9]–[11], to support conservation planning and reserve selection [12], and to evaluate the impact of global changes on species distributions [13], [14]. At a population level, SDMs have been used to estimate reproductive parameters [15] and/or population abundance [16]. Like other techniques in ecology and biogeography, SDMs require a theoretical framework that establishes relationships among different traits involved in the model (extent of the study area, data on species distribution, predictors, algorithms, among others) and the interpretation of the results [17]. In this respect, a relevant conceptual framework developed by Soberón and Peterson [18], and reviewed in later studies [3], can be used to obtain different types of models as a function of the part of the ecogeographical world of the modelled species. These inferences are relevant for applied ecology and conservation biology, and are particularly useful for the study of poorly known species.

In this context, here we study the Iberian hare (*Lepus granatensis* Rosenhauer, 1856), a species endemic to the Iberian Peninsula (southwestern Europe), where it plays an important socioecological role [19]. It is an important game species with more than 900,000 hares being harvested annually in Spain (http://www.marm.es/es/estadistica/temas/estadisticas-ambientales/biodiversidad2.aspx), and it has a significant role as prey for a large number of predators, such as the endangered Spanish imperial eagle (*Aquila adalberti*), especially in southern areas where the European rabbit (*Oryctolagus cuniculus*) has become scarce due to viral diseases [20], [21]. Notwithstanding its importance, data on the ecology of the Iberian hare in the international literature is scarce, since most information has been published in regional journals, books or reports with limited access. Yet, some studies focused on several aspects are available, as on reproduction [22], [23], parasites and health status [24], population size and dynamics [25], [26], diet [27], and habitat characteristics [24]–[26]. Also, particular attention has been given to the Iberian hare as a model to study speciation and reticulate evolution [31]–[36]. A striking pattern of mitochondrial DNA (mtDNA) introgression from the mountain hare (*L. timidus*) has been found in some populations of the Iberian hare, resulting from ancestral hybridization between these two species [31], [32]. This finding is remarkable, since the introgressed haplotypes are widespread through northern and central Iberian Peninsula, reaching massive frequencies in northern populations, albeit the mountain hare became locally extinct from the Iberian Peninsula, presumably at the end of the last glacial period as suggested by the fossil record [37]. Currently the mountain hare is found in northern Eurasia and in some isolated populations, such as Ireland, Scotland and the Alps [38]. Several studies have addressed the causes of such massive mtDNA introgression, and suggested that both demographic and selective processes may have contributed to create the current geographic patterns of genetic variation [34], [35]. However, clear inferences regarding the evolutionary history of the Iberian hare have often been hampered by poor knowledge on the species distribution and dynamics in critical periods such as at the last glacial maximum (LGM). As Ricklefs [39] and Levin [2] pointed out, local populations are also affected by historical and environmental processes that act at larger spatial scales. The study of large-scale processes is, therefore, important to complement knowledge on ecological studies conducted at local scales [40], stimulate the formulation of hypothesis on the ecological mechanisms modulating population dynamics [41] and also to guide local conservation programs [42].

Here we used data on the present natural-distribution of the Iberian hare and an appropriate theoretical framework to evaluate the relationship between this endemic species and the environment at large spatial scales (i.e., its macroecology) in order to determine its current ecogeographical correlates, complement the molecular information on its evolutionary history, and to assess its sensitivity to climate changes in the coming decades.

## Materials and Methods

### Study Area and Species Distribution Data

The main study area was the Iberian Peninsula, a biodiversity hotspot situated in south-western Europe. It is composed by the mainland territories of Portugal (approximately 15%) and Spain (approximately 85%), and has nearly 600,000 km^2^. The Iberian Peninsula is a discrete biogeographic unit since the Pyrenees cross the contact zone between the peninsula and the rest of Europe thereby limiting biotic and abiotic interactions.

**Figure 1:**
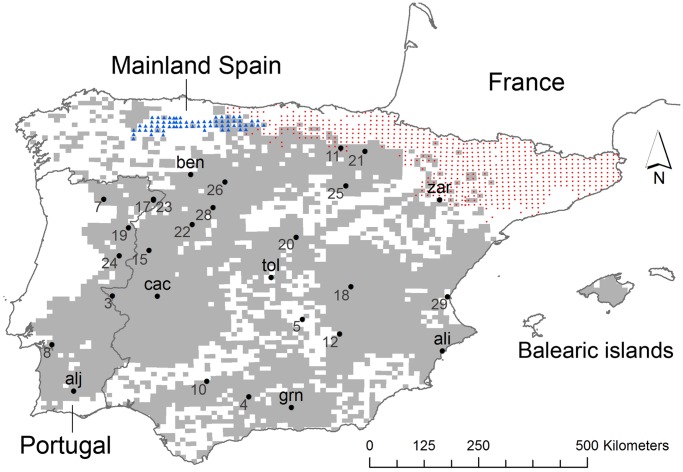
Distribution of *Lepus* sp. in the Iberian Peninsula. Current distribution of *Lepus granantensis* represented in UTM 10×10 km – grey – squares. Distribution ranges for sympatric hares species *L. castroviejoi* (blue triangles) and *L. europaeus* (red circles) in Spain are also displayed. Data were obtained from Almeida et al. [44] for Portugal and Palomo et al. [19] for Spain. Localities considered in the post-glacial colonization analyses (black circles) are also shown (data obtained from Tables S1 and S2 in Melo-Ferreira et al. [35]).

The Iberian hare is distributed throughout the Iberian Peninsula, as well as in southern France due to some local releases carried out for hunting purposes more than 20 years ago [43]. Species distribution data on UTM 10 km × 10 km grid squares (our territorial unit for modelling purposes) are available for Spain [19]. We updated this information for the Iberian Peninsula by transferring the hunting bag data available for Portugal [44] to our territorial units. This was done by considering as presences the squares in which individuals had been hunted in at least the half of their surface. This may be considered a conservative cut-off, but exploratory analyses carried out using 25% (instead of 50%) showed quite similar results in the distribution of the species in Portugal; only 10 localities (up to 875 presences within Portugal) changed their status (data not shown). We modelled the updated natural distribution of the Iberian hare (see Figure 1; 3519 presences up to 6256 territorial units) and excluded from the calibration datasets the non-native range of the species in order to avoid misinterpretations of the species’ macroecological requirements modulated by the allochthonous population [45].

### Environmental Predictors

A total of 39 ecogeographical variables related to several factors − spatial (2 variables), topoclimate (20 variables), land-cover (15 variables) and the distribution of other hare species occurring in parapatry in the study area (2 variables) − were used as predictors to model the current Iberian hare distribution range (see Table 1). The variables of the spatial factor (longitude and latitude) were considered to reveal the geographical trends in the species distribution, which are associated with historical events or species population dynamics [46]. In addition, a better fit to the species ranges can be obtained by including spatial variables as predictors, since these variables force spatial cohesion independently of the spatial distribution of environmental favourability [47]. Thus, these variables are needed to transfer distribution models to future periods [48].

**Table 1:** Variables used in the different models to study the *Lepus granatensis* distribution – past (P), present [explanatory] (E) and future (F) models – in the Iberian Peninsula.

Factor (model)	Codes	Description (units)
Spatial (E, F)	LAT	Latitude (decimal degrees)
	LONG	Longitude (decimal degrees)
Topoclimatic (P, E, F)	ALT	Altitude (masl)
	BIO1	Annual mean temperature (°C*10)
	BIO2	Mean diurnal range (mean of monthly [max T - min T]) (°C*10)
	BIO3	Isothermality (BIO2/BIO7) (*100)
	BIO4	Temperature seasonality (standard deviation*100)
	BIO5	Max temperature of warmest month (°C*10)
	BIO6	Min temperature of coldest month (°C*10)
	BIO7	Temperature annual range (BIO5–BIO6) (°C*10)
	BIO8	Mean temperature of wettest quarter (°C*10)
	BIO9	Mean temperature of driest quarter (°C*10)
	BIO10	Mean temperature of warmest quarter (°C*10)
	BIO11	Mean temperature of coldest quarter (°C*10)
	BIO12	Annual precipitation (mm)
	BIO13	Precipitation of wettest month (mm)
	BIO14	Precipitation of driest month(mm)
	BIO15	Precipitation seasonality (coefficient of variation)
	BIO16	Precipitation of wettest quarter (mm)
	BIO17	Precipitation of driest quarter (mm)
	BIO18	Precipitation of warmest quarter (mm)
	BIO19	Precipitation of coldest quarter (mm)
Land cover (E)	T11	Post-flooding or irrigated croplands (or aquatic) (%)
	T14	Rainfed croplands (%)
	T20	Mosaic cropland/vegetation (grassland/shrubland/forest) (%)
	T30	Mosaic vegetation/cropland (%)
	T50	Closed (>40%) broadleaved deciduous forest (%)
	T70	Closed needleleaved evergreen forest (%)
	T90	Open (15–40%) needleleaved deciduous or evergreen forest (%)
	T100	Closed to open (>15%) mixed forest (%)
	T120	Mosaic grassland/forest or shrubland (%)
	T130	Closed to open shrubland (%)
	T140	Closed to open herbaceous vegetation (grassland, savannas or lichens/mosses) (%)
	T150	Sparse (<15%) vegetation (%)
	T180	Closed to open grassland or woody vegetation on regularly flooded or waterlogged soil - Fresh, brackish or saline water (%)
	T200	Bare areas (%)
	T210	Water bodies (%)
*Lepus* spp. (E)	LEPEUR	Presence/absence of *L. europaeus* (categorical)
	LEPCAS	Presence/absence of *L. castroviejoi* (categorical)

A quarter is a period of three months (1/4 of the year).

The relevance of the topoclimatic factor to explain species distribution and abundance at large spatial scales is well known [49]. Data on bioclimatic variables and altitude are available in the Worldclim project database [50] and were downloaded from http://www.worldclim.org (∼1000 m spatial resolution). Assuming that the climatic requirements of the species have remained stable over time [51], the models calibrated on present distributions (see below) can be extrapolated to the past or future to identify the past and future environmental favourability for the species. This was done by replacing the current bioclimatic variables in the models with those estimated for the LGM ∼ 21,000 ybp [52], [53], available at ∼5000 m spatial resolution, and according to the CCCMA model and A2 emission scenario [54], for the future period (up to 2080; ∼1000 m spatial resolution). The selected emissions scenario represents an intermediate position regarding the wide range of projected shifts in temperature and precipitation [55].

We included land cover variables as predictors, in line with previous studies [28], [30]. Land cover data were taken from Global Land Cover 2005 (∼300 m spatial resolution), freely available at http://ionia1.esrin.esa.int/. This map has been designed and validated to be consistent at the global scale [56].

Finally, as the inclusion of biotic interactions improves the performance of the models [57], the distribution data of other hare species occurring in parapatry in the study area (broom hare [*L. castroviejoi*] and brown hare [*L. europaeus*]; data obtained from [19]) were also considered as predictors.

### Species Distribution Models: Current Distribution and Model Transferability

Using an inductive approach we determined the macroecological requirements of the Iberian hare based on the locations in which it occurred. Predictors were considered in a multiple logistic regression analysis [58], and the final models were obtained using a forwards-backwards stepwise procedure. Each model was calibrated using a 70% random sample of the species distribution data and evaluated against the remaining 30% of the data. Sensitivity (*Se*; the ratio of correctly predicted presences to the total number of presences), specificity (*Sp*; the ratio of correctly predicted absences to the total number of absences), and the AUC (the area under the receiver operating characteristic [ROC] curve) were computed to assess the discriminatory power of the models. To calculate *Se* and *Sp*, the continuous variables generated by models (predicted probabilities) were converted to a binary variable (presence-absence) selecting a cut-off point that minimizes the difference between *Se* and *Sp* [59]. Calibration of the probability values was assessed using the calibration plot, by plotting the proportion of evaluation sites found to be occupied for the species within each of ten equi-interval predicted probability classes, and thus perfect adjust points should lie along a 45° line (for details see [60]).

We parameterized three models for the Iberian hare pursuing different objectives with each one. First, we developed a model aimed at determining the requirements of the current species distribution (herein named “explanatory model”). The predictors considered in this model are shown Table 1. The model was partitioned to enhance its explanatory capacity and improve the reliability and interpretation of the model taking onto account multicollinearity between predictors [61]. Briefly, variation partitioning procedures are used to estimate the variation of the final model independently explained by each factor (pure effects) and the variation simultaneously explained by two or more factors (overlaid effects) following subtraction techniques [62]. For details on the subtraction techniques used in this study see, for example, Acevedo et al. [63].

A second model was designed to be hindcasted to the LGM (herein named “past model”). For this purpose, only climatic variables were used as predictors since i) no reconstructions for land cover during that period or data on species distributions are available, and ii) the spatial predictors should not be directly included because the spatial inertia of the species distribution cannot conceptually be extrapolated backward. A representation of the climatic potential for the species in the mentioned period was obtained by hindcasting this model [64].

Finally, we developed a model to be transferred to the future (herein named “future model”) to predict the effects of ongoing climate change on the current species distribution. In this case, and consistent with previous studies, the climatic variables and those related to the spatial factor were considered as predictors [48]. We excluded land cover variables since they are highly variable over time [65] and considered that current land cover is unlikely to remain the same in 2080. The model was projected to southwestern Europe to forecast the potential role of the adjacent territory to the current natural distribution – France – in relation to the future of the Iberian hare. We do not consider the distribution of other species since, to our best knowledge, data on the brown hare distribution at the UTM 10×10 km scale is not available for France. In addition, the results of a previous study [10] suggested that the distribution of the brown hare is predicted to have a reduced impact in limiting the Iberian hare occurrence.

To transfer models between time periods (required for both past and future models) and between territories (required to explore the future of the study species outside the Iberian Peninsula), multicollinearity between predictors should be controlled to avoid biased results [66], [67]. Thus, we checked the variance inflation factor (VIF) of each predictor to quantify collinearity between the predictors included in the final models. VIF is a positive value representing the overall correlation of each predictor with all others in a model and is calculated for each predictor as the inverse of the coefficient of non-determination for a regression of that predictor on all others [68]. We ensured that the selected predictors did not achieve a VIF >10 [69], [70].

### Species Potential Distribution for LGM and Postglacial Colonization

Previous phylogeographical studies on Iberian hare derived from native (non-introgessed) mtDNA reveal a lack of a clear geographical structure, despite the existence of three clear mtDNA lineages of mountain hare origin occurring in the Iberian Peninsula [35]. Nevertheless, a sub-lineage was discovered in central Iberia (Caceres region), which can reflect a refuge of the Iberia hare [35].

In this context, we used the genetic data reported in Melo-Ferreira et al. [35] to i) test the hypotheses of a central Iberia refugia and potential postglacial centrifugal colonization – i.e. an isotropic expansion from a given territory to the periphery –, and ii) evaluate the ecological meaning of the past model considering the molecular data for validation. Genetic differentiation (retrieved from mtDNA data available in [35]) among populations using the native mtDNA type only and measured as population pairwise Fst (with negative values fitted to zero) and Fst/(1−Fst) (see Table 2), calculated using Arlequin 3.5 [71] based on the pairwise sequence differences, were related with both the geographical proximities – i.e. straight-line geographic distances between pairs of populations – and the ecological distances among populations, the latter obtained from using the outputs of the past model. We computed the ecological distance between populations using the least-cost distance algorithm implemented in ArcGIS 10 [72]. This algorithm calculates a deterministic least-cost distance between a source population and a target population using a friction layer. Locations of sampled hare populations (Figure 1) were used in conjunction with a friction map representing the “cost of movement” through the landscape, i.e., the relative difficulty of moving through a given cell for the species. Least-cost distance minimizes the sum of movement costs of all cells along the path. Here, the friction map was obtained as one minus the predictions of the past model for the LGM.

**Table 2:** Genetic differentiation (index 1/index 2) retrieved from mitochondrial DNA (mtDNA) data available in Melo-Ferreira et al. [35], between each population and the studied potential refugia (localities coded as in Figure 1) using the native mtDNA type only and measured as population pairwise Fst (with negative values fitted to zero; index 1) and Fst/(1−Fst) (index 2).

Localities	Potencial refugia						
	ali	alj	ben	cac	grn	tol	zar
**alj**	0.38/0.60	NA	0.45/0.82	0.48/0.92	0.62/1.65	0.32/0.46	0.51/1.04
**cac**	0.48/0.92	0.48/0.92	0.31/0.46	NA	0.56/1.30	0.51/1.02	0.43/0.75
**3**	0.52/1.08	0.42/0.72	0.56/1.28	0.53/1.14	0.74/2.77	0.46/0.84	0.63/1.71
**4**	0.21/0.27	0.19/0.23	0.20/0.24	0.25/0.34	0.44/0.80	0.24/0.31	0.18/0.22
**5**	0.22/0.28	0.22/0.28	0.44/0.79	0.45–0.83	0.58/1.36	0.20/0.25	0.41/0.69
**grn**	0.57/1.35	0.62/1.65	0.52–1.08	0.56/1.30	NA	0.62/1.65	0.69/2.22
**7**	0.43/0.75	0.43/0.75	0.16/0.18	0.47/0.88	0.53/1.12	0.48/0.91	0.45/0.82
**8**	0.48/0.94	0.16/0.19	0.55/1.21	0.53/1.13	0.72/2.61	0.42/0.73	0.70/2.31
**10**	0.37/0.60	0.29/0.41	0.34/0.52	0.38/0.61	0.55/1.24	0.41/0.70	0.35/0.54
**11**	0.62/1.66	0.69/2.23	0.61/1.57	0.61/1.55	0.75/2.98	0.70/2.30	0.84/5.14
**12**	0.13/0.15	0.21/0.27	0.40/0.66	0.44/0.78	0.55/1.22	0.25/0.34	0.34/0.52
**ali**	NA	0.38/0.60	0.45/0.81	0.48/0.92	0.57/1.35	0.39/0.63	0.46/0.86
**15**	0.42/0.73	0.40/0.68	0.47/0.89	0.50/0.99	0.68/2.12	0.40/0.68	0.51/1.05
**ben**	0.45/0.81	0.45/0.82	NA	0.31/0.46	0.52/1.08	0.48/0.93	0.32/0.46
**17**	0.34/0.51	0.27/0.37	0/0	0.32/0.48	0.57/1.34	0.33/0.49	0.29–0.40
**18**	0.64/1.76	0.54/1.16	0.69/2.25	0.63/1.72	0.79/3.73	0.36/0.56	0.84/5.09
**19**	0.37/0.59	0.36/0.56	0.38/0.62	0.43/0.75	0.69/2.19	0.35/0.54	0.53/1.12
**20**	0.40/0.66	0.42/0.71	0.58/1.39	0.55/1.23	0.73/2.65	0.40/0.67	0.71/2.3
**21**	0.34/0.52	0.36/0.57	0.17/0.21	0.33/0.48	0.73/2.70	0/0	1.00/1.00
**22**	0.36/0.57	0.42/0.72	0.30/0.43	0.45/0.82	0.52/1.09	0.47/0.89	0.45/0.81
**23**	0.43/0.75	0.43/0.76	0.10/0.11	0.36/0.55	0.49/0.97	0.48/0.92	0.41/0.69
**24**	0.33/0.48	0.31/0.45	0.30/0.43	0.41/0.70	0.52/1.10	0.35/0.54	0.25/0.33
**25**	0.42/0.72	0.44/0.78	0.51/1.05	0.50/1.00	0.72/2.63	0.37/0.58	0.61/1.57
**26**	0.40/0.68	0.44/0.79	0.27/0.37	0.42/0.71	0.52/1.08	0.48/0.94	0.44/0.77
**tol**	0.39/0.63	0.32/0.462	0.48/0.93	0.51/1.02	0.62/1.65	NA	0.47/0.90
**28**	0.44/0.79	0.509/1.02	0.24/0.31	0.49/0.97	0.54/1.17	0.54/1.19	0.53/1.12
**29**	0.29/0.41	0.38/0.62	0.46/0.84	0.46/0.86	0.70/2.30	0.38/0.61	0.65/1.84
**zar**	0.46/0.86	0.51/1.04	0.32/0.46	0.43/0.75	0.69/2.22	0.47/0.90	NA

Past model for the LGM was used as a basis on which to hypothesize the location of potential glacial refugia. Concretely, we tested 7 localities (see Figure 1), and also combinations of significant localities, as potential refugia assuming a postglacial centrifugal colonization, as suggested by Melo-Ferreira et al. [35]. The ecological meaning of the predicted model for the LGM was validated by testing whether the genetic dissimilarity of each sampled population to a given potential refugium were better explained by the ecological distances than by geographical ones. For this purpose we used general lineal models and adopted the Akaike information Criteria for model comparisons (AIC; [73]).

## Results

### Ecogeographical Requirements of the Iberian Hare: Explanatory Model

**Figure 2:**
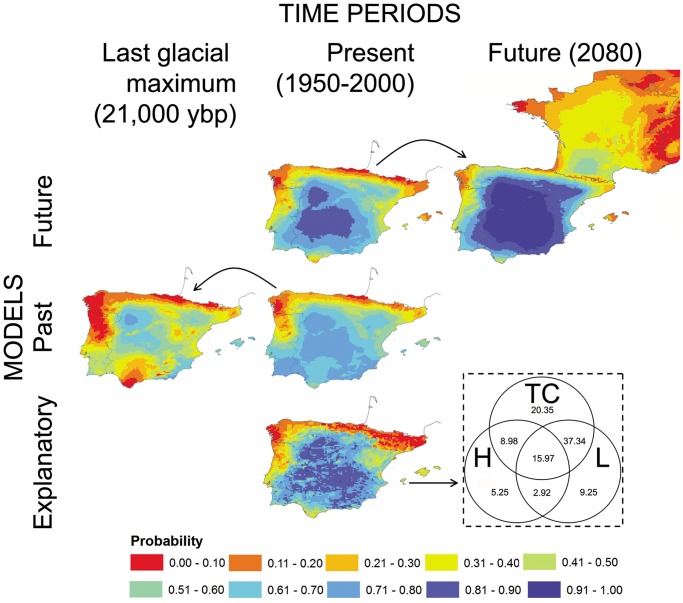
Cartographic representation of the statistical models. Probability of *Lepus granatensis* occurrence in the Iberian Peninsula obtained from the different models (see Table 3). Arrows indicates the transference of the models to the past or future (A2 emissions scenario) time periods. Variation partitioning of the explanatory model is shown in the inset. Values in the diagrams are the percentages of variation in hare presence explained exclusively by topoclimate (TC), land-cover (L), other parapatric *Lepus* spp. (H) and by the combined effect of these factors.

**Figure 3:**
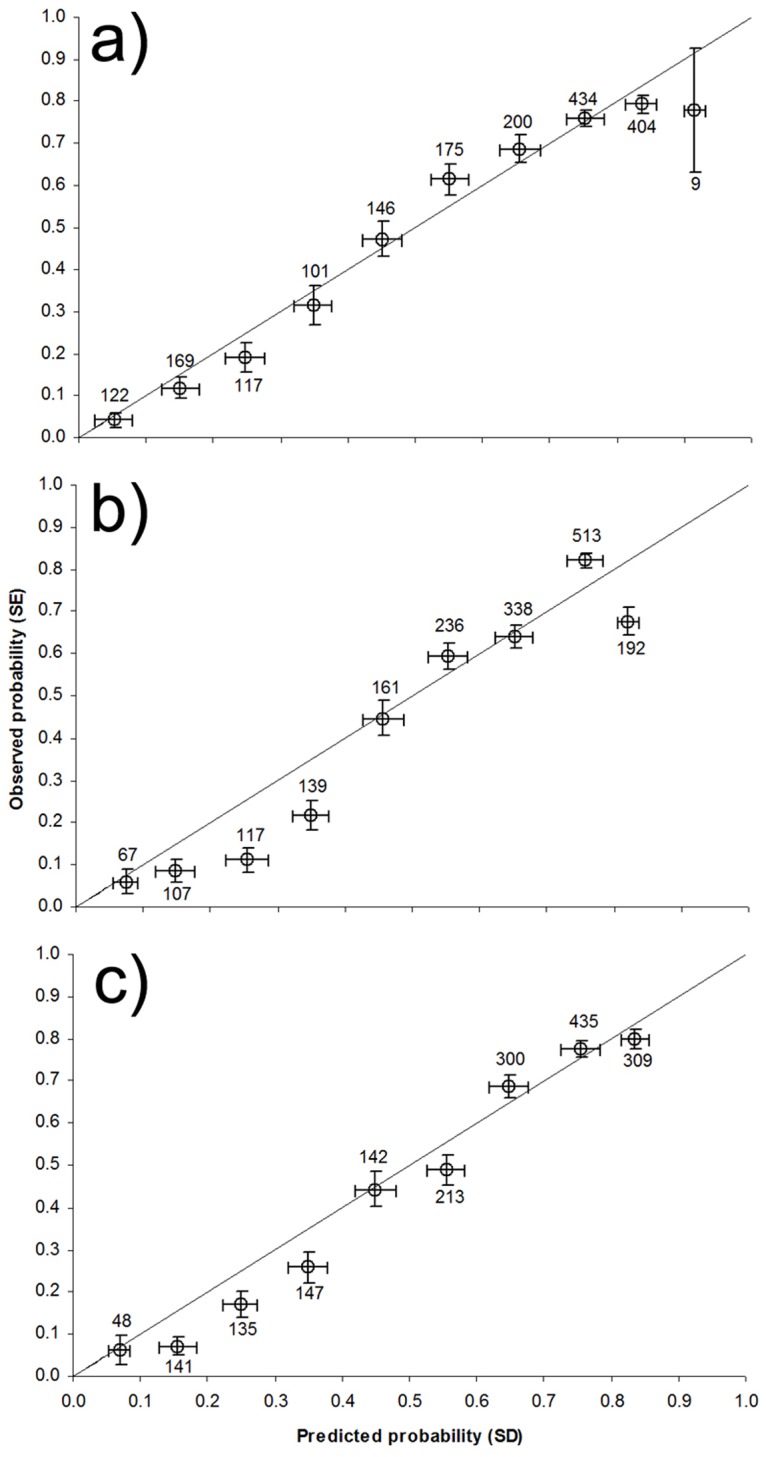
Calibration assessment of the statistical models. Calibration plots showing the relationship between the predicted probability of occurrence for the models and the observed proportion of evaluation localities currently occupied by *Lepus granatensis*: a) explanatory model (see Table 3a), b) model hindcasting to the past (see Table 3b) and c) model to be extrapolated to the future (see Table 3c). Numbers represent the number of evaluation localities in each interval of probability.

Only three factors were included in the explanatory model since the spatial factor was not retained (Table 3). Probability for the Iberian hare occurrence is widely distributed in the study area, and only northern and northwestern regions were predicted as highly improbable for the species (Figure 2). The variation partitioning procedure shows that the highest amount of variation can be explained by the topoclimatic factor, both its pure effect alone and the overlaid effect when it is combined with the other two factors (Figure 2). The model’s predictions achieved good performance when they were evaluated on an independent dataset in terms of discrimination (AUC: 0.79, Se: 0.71, and Sp: 0.71) and calibration (see Figure 3).

**Table 3:** Results of the explanatory model developed on the current distribution of *Lepus granatensis* (a), statistical models obtained for hindcasting to the past (b) and for extrapolation to the future (c) to predict the range of *L. granatensis* potentiality in these time periods.

Variable	*B*	SE	Wald	Sig.
a) Explanatory				
*Constant*	*−4.402*	*0.436*	*101.886*	***
T14	0.019	0.003	44.476	***
T20	0.020	0.003	43.184	***
T70	0.006	0.003	4.375	*
T90	−0.201	0.083	5.892	*
T120	0.048	0.007	44.629	***
T130	0.017	0.004	24.351	***
T150	0.022	0.004	37.356	***
ALT	0.001	<0.001	25.795	***
BIO2	0.016	0.004	20.620	***
BIO9	0.005	0.001	21.838	***
BIO13	−0.020	0.004	21.086	***
BIO19	0.004	0.001	7.175	**
LEPEUR	−1.501	0.180	69.309	***
b) Past model				
*Constant*	2.869	0.325	78.042	***
BIO1	−0.014	0.002	40.005	***
BIO13	−0.021	0.002	128.653	***
BIO14	−0.023	0.007	12.189	***
BIO15	0.035	0.007	23.872	***
c) Future model				
*Constant*	*−0.784*	*0.377*	*4.330*	*
X	−0.168	0.018	87.672	***
BIO2	0.009	0.005	3.197	*
BIO4	0.001	<0.001	37.377	***
BIO13	−0.016	0.001	116.125	***
BIO14	−0.017	0.003	29.744	***

*B* parameter coefficient and its standard error (*SE*), *Wald* Wald test statistics, *Sig*. significance (*<0.05, **<0.01 and ***<0.001). Variables coded as in Table 1.

### Hindcasting the Past Model and Linking it to the Postglacial Colonization

The past model (Table 3) suggests that the climatic favourability for the species is currently less restricted than it was 21,000 ybp (Figure 2). The past model’s predictions also achieved an acceptable predictive performance when they were evaluated on an independent dataset in terms of discrimination (AUC: 0.76, *Se*: 0.70, and *Sp*: 0.71) and calibration (see Figure 3). The selected predictors did not achieve a VIF>10 (range from 3.97 to 6.06).

Results on the assessment of potential glacial refuges in the Iberia Peninsula are summarized in Table 4. Only the localities Benavente (ben) and Aljustrel (alj) can be suggested as potential refugia if a centrifugal postglacial colonization is assumed. In addition, the overall ecological distances attained a higher explanatory capacity (lower AIC values) than the geographical distances in the analyses.

**Table 4:** Results of the linear regressions carried out to relate the genetic differentiation to a given population (potential refugium) and the geographical and ecological distances in order to test potential glacial refugia and postglacial centrifugal colonization.

Potential refugia	Genetic differentiation index	Geographical distance	Ecological distance
***ali***	*Fst*	0.229 ns	0.205 ns
	*Fst/(1*−*Fst)*	0.052 ns	0.033 ns
***alj***	*Fst*	−36.3/0.435*	−37.3/0.398*
	*Fst/(1*−*Fst)*	32.2/0.392*	31.1/0.355*
***ben***	*Fst*	−20.1/0.429*	−21.2/0.462*
	*Fst/(1*−*Fst)*	42.1/0.322 ns	41.8/0.372*
***cac***	*Fst*	0.063 ns	0.121 ns
	*Fst/(1*−*Fst)*	0.099 ns	0.153 ns
***grn***	*Fst*	0.232 ns	0.166 ns
	*Fst/(1*−*Fst)*	0.199 ns	0.125 ns
***Tol***	*Fst*	0.055 ns	0.019 ns
	*Fst/(1*−*Fst)*	0.103 ns	0.026 ns
***zar***	*Fst*	−11.7/−0.425*	−10.1/−0.361 ns
	*Fst/(1*−*Fst)*	87.7/−0.387 ns	89.2/−0.314 ns
***alj-ben***	*Fst*	−20.2/0.511**	−21.2/0.512**
	*Fst/(1*−*Fst)*	41.2/0.428*	40.9/0.440*
***ben-cac***	*Fst*	0.374 ns	0.364 ns
	*Fst/(1*−*Fst)*	0.337 ns	0.338 ns
***alj-cac***	*Fst*	0.200 ns	0.233 ns
	*Fst/(1*−*Fst)*	0.216 ns	0.238 ns
***ben-alj-cac***	*Fst*	0.388 ns	0.388 ns
	*Fst/(1*−*Fst)*	0.381 ns	0.372 ns
***alj-ben-zar***	*Fst*	0.313 ns	0.379 ns
	*Fst/(1*−*Fst)*	0.192 ns	0.275 ns
***alj-ben-ali-zar***	*Fst*	0.292 ns	0.402 ns
	*Fst/(1*−*Fst)*	0.216 ns	0.325 ns

Values are the AIC [73]/Pearson coefficient and significance (*<0.05, **<0.01 and ***<0.001). Only for significant regressions the AICs are reported. Localities are coded as in Figure 1.

### Forecasting Iberian hare Distributions in the Future

The model transferred to the future retained both spatial and climatic variables (Table 3). The probability of the Iberian hare occurrence is expected to increase in the future within the current species range and, to a lesser extent, a northward shift in the species distribution limit was also forecast (Figure 2). As in the other models presented here, the predictive performance of the future model was acceptable when it was assessed on an independent dataset, both in terms of discrimination (AUC: 0.78, *Se*: 0.72, and *Sp*: 0.72) and calibration (see Figure 3). The selected predictors did not achieve a VIF >10 (range from 2.67 to 6.62).

## Discussion

### Factors Driving Current Iberian Hare Distribution

This study constitute the first comprehensive work on the ecological requirements of the Iberian hare, not only in relation to its geographical context – the entire native distribution area of the species was considered – but also regarding the broad set of predictors of its distribution range. To our knowledge, the few available studies were focused on the ecological determinants at a regional level [28]–[30]. The overall distribution of the species has to be considered when the aim is to assess the response of the species to the entire range of ecogeographical gradients [74]. Similarly, the actual weight of each factor can only be precisely determined by including a wide set of potential factors driving the species distribution [48]. Therefore, distribution models requiring the estimation of borders or their ecogeographical correlates may fail if only local data or a narrow set of factors are used [75].

Our explanatory model shows that the Iberian hare distribution is mainly modulated by the topoclimatic factor when its pure effect is considered and when topoclimate is combined with land cover. The high relevance of topoclimate factor is not surprising given the spatial scale of this study and that the Iberian hare distribution is linked to the Mediterranean climate in the Iberian Peninsula [76].

The Iberian hare strongly depends on land cover according to previous studies conducted in the northwestern and southern Iberian Peninsula [28]–[30]. The species dependence on land cover is highly relevant for conservation since, in contrast to climate, it is possible to manage land cover for species conservation. Based on our results, management strategies aiming at improving the habitat of the Iberian hare can be proposed as for example the conservation of open Mediterranean scrubland and habitat heterogeneity in the agroecosystems [30]. It should be noted here that even when coarse-resolution distribution models are useful to guide species conservation programs [42], sometimes management requires finer spatial scales than those captured by these kind of approaches [77].

The distribution of the brown hare was also retained in the explanatory model since this species occurs in parapatry with the Iberian hare in northern Iberia. Thus, this study provides evidence on the role of interspecific – parapatric – interactions in determining species distribution ranges [10]. To understand this concrete relationship it should be pointed out that where the Iberian hare and the brown hare coexist in Iberia, some advantages of the Iberian hare over the brown hare were suggested from a macroecological study [10]. Thus, the distribution of the Iberian hare – even in parapatry – is not expected to be highly constrained by interspecific relationships with the brown hare.

Finally, but of no less importance, it should be emphasized that the model’s output is a reliable index of the local probability of species occurrence as evidenced from the calibration plot (Figure 3). Thus, the statistical model and the derived map shown in Figure 2 are valuable tools to guide species management and conservation planning at a global spatial scale [42], [78], [79].

### Past Distribution of the Iberian Hare: Postglacial Colonization

The hindcast of SDMs to LGM conditions has helped to determine the climatic potential for the species in key periods in relation to the species evolutionary history. In this context, SDMs are very valuable tools to assist in understanding phylogeographical hypotheses derived from molecular ecology studies [80], [81]. The results obtained in this study allow reinterpreting previous suggestions regarding the range dynamics of the Iberian hare during the LGM based on population genetics approaches. The molecular inference shows i) a south-north gradient of increasing frequencies of introgressed mtDNA haplotypes of the mountain hare origin in populations of the Iberian hare, ii) a northward increase in haplotype diversity among the introgressed haplotypes, and iii) sectors of differentiation perpendicular to the introgression limits, which is compatible with introgression occurring during the range replacement of the mountain hare by the Iberian hare after the LGM ([32], [33], [35]; for a theoretical discussion see [82]). This hypothesis implies that the Iberian hare recently colonized northern Iberia, the area where the mountain hare was presumably present at the end of the last glacial period as suggested by the fossil record [37]. However, the presence of mountain hare variants in southern Iberian hare populations detected in the analyses of autosomal [34] and X-linked markers [35] has challenged this simplified hypothesis and has led to the suggestion that the Iberian hare may have also recently colonized the south from a central Iberian glacial refugium. This centrifugal colonization hypothesis is also supported by the inference of the highest diversity among the Iberian hare mtDNA haplotypes in the population in Caceres in central Iberia [35]. Interestingly, this region is also depicted in this study as a climatically suitable area for the species during the LGM (Figure 2). However, other areas in the north and south are also suggested as potential suitable areas, which may have also acted as attractive areas determining the post-glacial colonization routes of the Iberian hare. We here tested several regions as potential refuge for the Iberian hare at LGM and our results showed significant correlations when Aljustrel or Benavente, or both, were assessed as refugia (Figure 3). Considering these populations as references, the genetic isolation by distance is maximized which may indicate that these geographical areas (separately or together) may have been the centre of postglacial colonization of Iberia. If this is the case, the hypothesis of a recent centrifugal colonization of Iberia by Iberian hare from multiple refugia is supported by our results. Interestingly, the ecological distances, in general, performed better in explaining the genetic differentiation, which suggest that the model obtained for LGM is meaningful, i.e., the climatic favourability for the species may have been an important determinant of postglacial colonization routes.

### Expected Impact of Climate Change on Iberian Hare Distribution

The predictions obtained here for the Iberian hare distribution in 2080 contrast with other studies, in which endemic species are predicted to be strongly negatively affected by future climatic changes, mainly in Spain and North Africa [83], [84]. The Iberian hare seems to be an exception to this general trend. Based on our study, not only this species is expected to expand its potential distribution towards the north-east – reaching moderate probabilities of occurrence in southern France – but also to achieve higher probability for species occurrence in its core distribution area. The forecasted pattern cannot be properly validated because of the lack of data concerning the future. However, it seems consistent with the expert knowledge on the species, since hot extremes in summer, together with the climate change predicted for the North which tends to fit better the general climatic requirements of the Iberian hare are expected in the Mediterranean region [85].

During the last decade, several studies have attempted to project SDMs into the future using a range of future climate change scenarios to evaluate the potential effects of future climate change on biodiversity [14]. Even though the usefulness of SDMs has been questioned, this task is best achieved by using modelling tools to complement expert assessments [86]. Thus, SDMs, if carefully implemented, are useful tools to assess the sensitivity of species to climate change, defined in this context as the degree to which their distributions are affected by climate change. Many studies have been conducted on an huge set of species in order to explore the global effects of climate changes on biodiversity [14]. Even though we acknowledge the relevance of these studies, the importance of analyses focused on a single species should be emphasized, since multi-species approaches are sometimes necessarily too simplistic or superficial – usually only climatic predictors are used [48] – when this kind of assessment ideally requires a complete determination of the environmental determinants of the species [13], [78].

### Conclusions

This study provides an example of how gaps in species macroecological knowledge can be filled using SDMs. This approach has several benefits. First, the use of SDMs in a contemporary setting helps understanding the role of the different factors modulating species distribution. Second, evaluating suitability in the past is useful to better understand the hypothesis of species evolution driven by phylogeographical studies [34]. Finally, the model for the future has enabled us to predict the impact of climate change on the species distribution, making it possible to devise a response from conservationists to the effects of climate change on species distribution. These aspects are particularly important when dealing with endemic and poorly-known species. Thus, this study shows how clues on species ecology and evolutionary history can be extracted from species distribution data and an appropriate conceptual framework.

## References

[1] Franklin J (2009) Mapping species distributions. Spatial inference and prediction. Cambridge: Cambridge University Press. 320 p.

[2] Levin SA (1992) The problem of pattern and scale in ecology. Ecology 73: 1943–1967.

[3] Soberón JM (2010) Niche and area of distribution modeling: a population ecology perspective. Ecography 33: 159–167.

[4] Guisan A, Thuiller W (2005) Predicting species distribution: offering more than simple habitat models. Ecol Lett 8: 993–1009. 10.1111/j.1461-0248.2005.00792.x 34517687

[5] Guisan A, Zimmermann NE (2000) Predictive habitat distribution models in ecology. Ecol Model 135: 147–186.

[6] Acevedo P, Alzaga V, Cassinello J, Gortázar C (2007) Habitat suitability modelling reveals a strong niche overlap between two poorly known species, the broom hare and the Pyrenean grey partridge, in the north of Spain. Acta Oecol 31: 174–184.

[7] Guisan A, Broennimann O, Engler R, Vust M, Yoccoz NG, et al (2006) Using niche-based models to improve the sampling of rare species. Conserv Biol 20: 501–511. 16903111 10.1111/j.1523-1739.2006.00354.x

[8] Peterson AT, Vieglais DA (2001) Predicting species invasions using ecological niche modeling: new approaches from bioinformatics attack a pressing problem. BioScience 51: 363–371.

[9] Acevedo P, Ward AI, Real R, Smith GC (2010) Assessing biogeographical relationships of ecologically related species using favourability functions: a case study on British deer. Divers Distrib 16: 515–528.

[10] Acevedo P, Jiménez-Valverde A, Melo-Ferreira J, Real R, Alves PC (2012) Parapatric species and the implications for climate change studies: a case study on hares in Europe. Global Change Biol 18: 1509–1519.

[11] Real R, Barbosa AM, Rodríguez A, García FJ, Vargas JM, et al (2009) Conservation biogeography of ecologically interacting species: the case of the Iberian lynx and the European Rabbit. Divers Distrib 15: 390–400.

[12] Estrada A, Real R, Vargas JM (2011) Assessing coincidence between priority conservation areas for vertebrate groups in a Mediterranean hotspot. Biol Conserv 144: 1120–1129.

[13] Brambilla M, Casale F, Bergero V, Bogliani G, Crovetto GM, et al (2010) Glorious past, uncertain present, bad future? Assessing effects of land-use changes on habitat suitability for a threatened farmland bird species. Biol Conserv 143: 2770–2778.

[14] Thuiller W, Lavergne S, Roquet C, Boulangeat I, Lafourcade B, et al (2011) Consequences of climate change on the tree of life in Europe. Nature 470: 531–534. 21326204 10.1038/nature09705

[15] Brambilla M, Ficetola GF (2012) Species distribution models as a tool to estimate reproductive parameters: a case study with a passerine bird species. J Anim Ecol 81: 781–787. 22372863 10.1111/j.1365-2656.2012.01970.x

[16] VanDerWal J, Shoo LP, Johnson CN, Williams SE (2009) Abundance and the environmental niche: environmental suitability estimated from niche models predicts the upper limit of local abundance. Am Nat 174: 282–291. 19519279 10.1086/600087

[17] Jiménez-Valverde A, Lobo JM, Hortal J (2008) Not as good as they seem: the importance of concepts in species distribution modelling. Divers Distrib 14: 885–890.

[18] Soberón J, Peterson AT (2005) Interpretation of models of fundamental ecological niches and species’ distribution areas. Biodiversity Informatics 2: 1–10.

[19] Palomo LJ, Gisbert J, Blanco JC (2007) Atlas y Libro Rojo de los mamíferos terrestres de España. Madrid: Ministerio de Medio Ambiente. 585 p.

[20] Delibes-Mateos M, Redpath SM, Angulo E, Ferreras P, Villafuerte R (2007) Rabbits as a keystone species in southern Europe. Biol Conserv 137: 149–156.

[21] Carro F, Soriguer RC (2010) La liebre ibérica. Madrid: Organismo Autónomo de Parques Nacionales. 364 p.

[22] Alves PC, Gonçalves H, Santos M, Rocha A (2002) Reproductive biology of the Iberian hare, *Lepus granatensis*, in Portugal. Mamm Biol 67: 358–371.

[23] Farfán MA, Vargas JM, Real R, Palomo LJ, Duarte J (2004) Population parameters and reproductive biology of the Iberian hare *Lepus granatensis* in southern Iberia. Acta Theriol 49: 319–335.

[24] Alzaga V, Vicente J, Villanua D, Acevedo P, Casas F, et al (2008) Body condition and parasite intensity correlates with escape capacity in Iberian hares (*Lepus granatensis*). Behav Ecol Sociobiol 62: 769–775.

[25] Gortázar C, Millán J, Acevedo P, Escudero MA, Marco J, et al (2007) A large-scale survey of brown hare *Lepus europaeus* and Iberian hare *L. granatensis* populations at the limit of their ranges. Wildlife Biol 13: 244–250.

[26] Lazo A, de le Court C, Soriguer RC (1992) Evaluation of the hare abundance allowed by their use of attraction point. Z Säugetierkd 7: 373–379.

[27] Paupério J, Alves PC (2008) Diet of the Iberian hare (*Lepus granatensis*) in a mountain ecosystem. Eur J Wildlife Res 54: 571–579.

[28] Vargas JM, Farfán MA, Guerrero JC, Barbosa AM, Real R (2007) Geographical and environmental correlates of big and small game in Andalusia (southern Spain). Wildlife Res 34: 498–506.

[29] Tapia L, Dominguez J, Rodríguez L (2010) Modelling habitat use by Iberian hare *Lepus granatensis* and European wild rabbit *Oryctolagus cuniculus* in a mountainous area in northwestern Spain. Acta Theriol 55: 73–79.

[30] Farfán MA, Duarte J, Vargas JM, Fa JE (2012) Effects of human induced land-use changes on the distribution of the Iberian hare. J Zool. doi:10.1111/j.1469–7998.2011.00873.x.

[31] Alves PC, Ferrand N, Suchentrunk F, Harris DJ (2003) Ancient introgression of *Lepus timidus* mtDNA into *L. granatensis* and *L. europaeus* in the Iberian Peninsula. Mol Phylogenet Evol 27: 70–80. 12679072 10.1016/s1055-7903(02)00417-7

[32] Melo-Ferreira J, Boursot P, Suchentrunk F, Ferrand N, Alves PC (2005) Invasion from the cold past: extensive introgression of mountain hare (*Lepus timidus*) mitochondrial DNA into three other hare species in northern Iberia. Mol Ecol 14: 2459–2464. 15969727 10.1111/j.1365-294X.2005.02599.x

[33] Melo-Ferreira J, Boursot P, Randi E, Kryukov A, Suchentrunk F, et al (2007) The rise and fall of the mountain hare (*Lepus timidus*) during Pleistocene glaciations: expansion and retreat with hybridization in the Iberian Peninsula. Mol Ecol 16: 605–618. 17257116 10.1111/j.1365-294X.2006.03166.x

[34] Melo-Ferreira J, Alves PC, Freitas H, Ferrand N, Boursot P (2009) The genomic legacy from the extinct *Lepus timidus* to the three hare species of Iberia: contrast between mtDNA, sex chromosomes and autosomes. Mol Ecol 18: 2643–2658. 19457181 10.1111/j.1365-294X.2009.04221.x

[35] Melo-Ferreira J, Alves PC, Rocha J, Ferrand N, Boursot P (2011) Interspecific X-chromosome and mitochondrial DNA introgression in the Iberian hare: selection or allele surfing? Evolution 65: 1956–1968. 21729051 10.1111/j.1558-5646.2011.01261.x

[36] Melo-Ferreira J, Boursot P, Carneiro M, Esteves PJ, Farelo L, et al (2012) Recurrent introgression of mitochondrial DNA among hares (*Lepus* spp.) revealed by species-tree inference and coalescent simulations. Syst Biol 61: 367–381. 22201159 10.1093/sysbio/syr114

[37] Altuna J (1970) Hallazgo de una liebre artica (*Lepus timidus*) en el yacimiento prehistorico de Urtiga (Guipuzcoa). Munibe 22: 165–168.

[38] Mitchell-Jones AJ, Amori G, Bogdanowicz W, Kryðtufek B, Reijnders PJH, et al.. (1999) The atlas of european mammals. London: T & AD Poyser Ltd. 250 p.

[39] Ricklefs RE (1987) Community diversity: relative roles of local and regional processes. Science 235: 167–171. 17778629 10.1126/science.235.4785.167

[40] Vaughn CC, Taylor CM (2000) Macroecology of a host-parasite relationship. Ecography 23: 11–20.

[41] Muñoz AR, Real R, Barbosa AM, Vargas JM (2005) Modelling the distribution of Bonelli’s eagle in Spain: implications for conservation planning. Divers Distrib 11: 477–486.

[42] Barbosa AM, Real R, Vargas JM (2010) Use of coarse-resolution models of species’ distributions to guide local conservation inferences. Conserv Biol 24: 1378–1387. 20455912 10.1111/j.1523-1739.2010.01517.x

[43] Smith AT, Johnston CH (2008) *Lepus granatensis* In: IUCN 2011. IUCN Red List of Threatened Species. Version 2011.1. Available: www.iucnredlist.org. Accessed 2012 Aug 1.

[44] Almeida J, Santos E, Bio A (2004) Characterization of population and recovery of Iberian hare in Portugal through direct sequential co-simulation. Quantitative Geol Geostat 13: 127–138.

[45] Acevedo P, Cassinello J (2009) Human-induced range expansion of wild ungulates causes niche overlap between previously allopatric species: red deer and Iberian ibex in mountainous regions of southern Spain. Ann Zool Fenn 46: 39–50.

[46] Real R, Barbosa AM, Porras D, Kin MS, Márquez AL, et al (2003) Relative importance of environment, human activity and spatial situation in determining the distribution of terrestrial mammal diversity in Argentina. J Biogeogr 30: 939–947.

[47] De Marco P Jr, Diniz-Filho JAF, Bini LM (2008) Spatial analysis improves species distribution modelling during range expansion. Biol Letters 4: 577–580. 10.1098/rsbl.2008.0210 PMC2610070 18664417

[48] Márquez AL, Real R, Olivero J, Estrada A (2011) Combining climate with other influential factors for modelling the impact of climate change on species distribution. Clim Change 1–23.

[49] Gutiérrez-Illán J, Gutiérrez D, Wilson RW (2010) The contributions of topoclimate and land cover to species distributions and abundance: fine resolution tests for a mountain butterfly fauna. Global Ecol Biogeogr 19: 15–173.

[50] Hijmans RJ, Cameron SE, Parra JL, Jones PG, Jarvis A (2005) Very high resolution interpolated climate surfaces for global land areas. Int J Climatol 25: 1965–1978.

[51] Heikkinen RK, Luoto M, Araújo MB, Virkkala R, Thuiller W, et al (2006) Methods and uncertainties in bioclimatic envelope modeling under climate change. Prog Phys Geog 30: 751–777.

[52] Otto-Bliesner BL, Marshall SJ, Overpeck JT, Miller GH, Hu A, et al (2008) Simulating arctic climate warmth and icefield retreat in the last interglaciation. Science 311: 1751–1753. 10.1126/science.1120808 16556838

[53] Braconnot P, Otto-Bliesner B, Harrison S, Joussaume S, Peterschmitt J-Y, et al (2007) Results of PMIP2 coupled simulations of the Mid-Holocene and Last Glacial Maximum - Part 1: experiments and large-scale features. Clim Past 3: 261–277.

[54] Nakicenovic N, Alcamo J, Davis G, de Vries B, Fenhann J, et al.. (2000) IPCC Special report on emissions scenarios. Cambridge: Cambridge University Press. 599 p.

[55] Brunet M, Casado MJ, de Castro M, Galán P, Lopez JA, et al.. (2007) Generación de escenarios de cambio climático para España. Ministerio de Medio Ambiente, Madrid.

[56] Bicheron P, Defourny P, Brockmann C, Schouten L, Vancutsem C, et al. (2008) “GlobCover 2005– Products description and validation report”, Version 2.1, 2008 (a). Available: http://ionia1.esrin.esa.int/. Accessed 2011 Aug 1.

[57] Araújo MB, Luoto M (2007) The importance of biotic interactions for modelling species distributions under climate change. Global Ecol Biogeogr 16: 743–753.

[58] Hosmer DW, Lemeshow S (1989) Applied logistic regression. New York: John Wiley and Sons, Inc. 307 p.

[59] Liu C, Berry PM, Dawson TP, Pearson RG (2005) Selecting thresholds of occurrence in the prediction of species distributions. Ecography 28: 385–393.

[60] Pearce J, Ferrier S (2000) Evaluating the predictive performance of habitat models developed using logistic regression. Ecol Model 133: 225–245.

[61] Graham MH (2003) Confronting multicollinearity in ecological multiple regression. Ecology 84: 2809–2815.

[62] Borcard D, Legendre P, Drapeau P (1992) Partialling out the spatial component of ecological variation. Ecology 73: 1045–1055.

[63] Acevedo P, Ruiz-Fons F, Estrada R, Márquez AL, Miranda MA, et al (2010) A broad assessment of factors determining *Culicoides imicola* abundance: modelling the present and forecasting its future in climate change scenarios. PLoS ONE 5 (12): e14236 doi:10.1371/journal.pone.0014236 10.1371/journal.pone.0014236 PMC2997795 21151914

[64] Nogués-Bravo D (2009) Predicting the past distribution of species climatic niches. Global Ecol Biogeogr 18: 521–531.

[65] Acevedo P, Farfán MA, Márquez AL, Delibes-Mateos M, Real R, et al (2011) Past, present and future of wild ungulates in relation to changes in land use. Landscape Ecol 26: 19–31.

[66] Barbosa AM, Real R, Vargas JM (2009) Transferability of environmental favourability models in geographic space: The case of the Iberian desman (*Galemys pyrenaicus*) in Portugal and Spain. Ecol Model 220: 747–754.

[67] Tuanmu M-N, Viña A, Roloff GJ, Liu W, Ouyang Z, et al (2011) Temporal transferability of wildlife habitat models: implications for habitat monitoring. J Biogeogr 38: 1510–1523.

[68] Zuur AF, Ieno EN, Elphick CS (2010) A protocol for data exploration to avoid common statistical problems. Methods Ecol Evol, 1, 3–14.

[69] Montgomery DC, Peck EA (1992) Introduction to Linear Regression Analysis. New York: Wiley. 672 p.

[70] O’Brien RM (2007) A caution regarding rules of thumb for variance inflation factors. Qual Quant 41: 673–690.

[71] Excoffier L, Lischer HEL (2010) Arlequin suite ver 3.5: A new series of programs to perform population genetics analyses under Linux and Windows. Mol Ecol Resour 10: 564–567. 21565059 10.1111/j.1755-0998.2010.02847.x

[72] ESRI (2011) ArcGIS Desktop: Release 10. Redlands, CA: Environmental Systems Research Institute.

[73] Akaike H (1974) A new look at the statistical model identification. IEEE Trans Autom Control 19: 716–723.

[74] Thuiller W, Brotons L, Araújo MB, Lavorel S (2004) Effects of restricting environmental range of data to project current and future species distributions. Ecography 27: 165–172.

[75] Sánchez-Fernández D, Lobo JM, Hernández-Manrique OL (2011) Species distribution models that do not incorporate global data misrepresent potential distributions: a case study using Iberian diving beetles. Divers Distrib 17: 163–171.

[76] Rivas-Martínez S, Penas A, Díaz TE (2004) Biogeographic map of Europe. Cartographic Service, University of León. Available: http://www.globalbioclimatics.org. Accessed 2011 Sep 1.

[77] Brambilla M, Casale F, Bergero V, Crovetto M, Falco R, et al (2009) GIS-models work well, but are not enough: habitat preferences of Lanius collurio at multiple levels and conservation implications. Biol Conserv 142: 2033–2042.

[78] Fouquet A, Ficetola GF, Haigh A, Gemmell N (2010) Using ecological niche modelling to infer past, present and future environmental suitability for *Leiopelma hochstetteri*, an endangered New Zealand native frog. Biol Conserv 143: 1375–1384.

[79] Wilson CD, Roberts D, Reid N (2011) Applying species distribution modelling to identify areas of high conservation value for endangered species: A case study using *Margaritifera margaritifera* (L.). Biol Conserv 144: 821–829.

[80] Hugall A, Moritz C, Moussalli A, Stanisic J (2002) Reconciling paleodistribution models and comparative phylogeography in the Wet Tropics rainforest land snail *Gnarosophia bellendenkerensis* (Brazier 1875). Proc Natl Acad Sci USA 99: 6112–6117. 11972064 10.1073/pnas.092538699 PMC122911

[81] Knowles LL, Alvarado-Serrano DF (2009) Exploring the population genetic consequences of the colonization process with spatio-temporally explicit models: insights from coupled ecological, demographic and genetic models in montane grasshoppers. Mol Ecol 19: 3727–3745. 10.1111/j.1365-294X.2010.04702.x 20723059

[82] Petit RJ, Excoffier L (2009) Gene flow and species delimitation. Trends Ecol Evol 24: 386–393. 19409650 10.1016/j.tree.2009.02.011

[83] Levinsky I, Skov F, Svenning J-C, Rahbek C (2007) Potential impacts of climate change on the distributions and diversity patterns of European mammals. Biodivers Conserv 16: 3803–3816.

[84] Maiorano L, Falcucci A, Zimmermann NE, Psomas A, Pottier J, et al (2011) The future of terrestrial mammals in the Mediterranean basin under climate change. Philos T R Soc B 366: 2681–2692. 10.1098/rstb.2011.0121 PMC3140741 21844047

[85] Gao X, Giorgi F (2008) Increased aridity in the Mediterranean region under greenhouse gas forcing estimated from high resolution simulations with a regional climate model. Global Planet Change 62: 195–209.

[86] Pearson RG, Dawson TP (2003) Predicting the impacts of climate change on the distribution of species: are bioclimate envelope models useful? Global Ecol Biogeogr 12: 361–371.

